# Spore germination in *Saccharomyces cerevisiae*: global gene expression patterns and cell cycle landmarks

**DOI:** 10.1186/gb-2007-8-11-r241

**Published:** 2007-11-14

**Authors:** Daphna Joseph-Strauss, Drora Zenvirth, Giora Simchen, Naama Barkai

**Affiliations:** 1 Departments of Molecular Genetics and Physics of Complex System, Weizmann Institute of Science, Rehovot 76100, Israel; 2 Department of Genetics, The Hebrew University of Jerusalem, Jerusalem 91904, Israel

## Abstract

**Background:**

Spore germination in the yeast *Saccharomyces cerevisiae *is a process in which non-dividing haploid spores re-enter the mitotic cell cycle and resume vegetative growth. To study the signals and pathways underlying spore germination we examined the global changes in gene expression and followed cell-cycle and germination markers during this process.

**Results:**

We find that the germination process can be divided into two distinct stages. During the first stage, the induced spores respond only to glucose. The transcription program during this stage recapitulates the general transcription response of yeast cells to glucose. Only during the second phase are the cells able to sense and respond to other nutritional components in the environment. Components of the mitotic machinery are involved in spore germination but in a distinct pattern. In contrast to the mitotic cell cycle, growth-related events during germination are not coordinated with nuclear events and are separately regulated. Thus, genes that are co-induced during G1/S of the mitotic cell cycle, the dynamics of the septin Cdc10 and the kinetics of accumulation of the cyclin Clb2 all exhibit distinct patterns of regulation during spore germination, which allow the separation of cell growth from nuclear events.

**Conclusion:**

Taken together, genome-wide expression profiling enables us to follow the progression of spore germination, thus dividing this process into two major stages, and to identify germination-specific regulation of components of the mitotic cell cycle machinery.

## Abstract 2

Genome-wide expression profiling of spore germination in Saccharomyces cerevisiae reveals two major stages and identifies germination-specific regulation of cell cycle machinery.

## Background

Spore germination in *Saccharomyces cerevisiae *is the process by which resting, non-dividing spores grow and enter the mitotic cell cycle. Mitotic cell cycle events are driven by a robust oscillatory system. This mitotic oscillator is regulated by a complex but well characterized network of regulatory proteins affecting transcription, protein phosphorylation and stability of activators and inhibitors [1-4]. However, cells are capable of exiting the cell cycle and entering a different, resting state. Only under appropriate conditions do the resting cells re-enter the mitotic cycle and resume growth and division. Thus, the mitotic oscillator controlling the cell cycle has to resume. In contrast to the well-studied vegetative cell cycle in yeast, and despite the importance of the resting stage to the life cycle of the cell, the mechanisms regulating entry, maintenance and exit from rest are poorly understood.

*S. cerevisiae *cells may enter into either of two resting states, namely stationary phase or spore formation. Diploid cells starved of both fermentable carbon and nitrogen sources leads to the formation of spores through the process of meiosis (which also involves reduction of chromosome number from diploid to haploid). Spores show unique characteristics and are more resistant to different environmental stresses than vegetative cells. The different processes of exit from rest (that is, spore germination and exit from stationary phase) share similar features, namely response to an extracellular signal and resumption of the mitotic cell cycle state. Therefore, it seems likely that the different transitions from quiescence to the mitotic cell cycle all share similar mechanisms. Thus, spore germination is not only an important process in the yeast life cycle, but studying this process may also deepen our understanding of other processes involved in exit from resting states.

It is thought that resting yeast cells re-enter the mitotic cycle through the G1 phase. However, not much is known about the involvement of the mitotic cell cycle machinery in exit from rest and particularly during spore germination. Most cell cycle regulators examined for their involvement in spore germination were not required for early stages of this process [5]. Nevertheless, the involvement of these proteins in later stages of germination, but before the mitotic cell cycle is entered, has not been examined.

Spore germination is initiated when nutrients are provided. Similar to the mitotic cell division cycle, spore germination is sustained by complete medium that contains carbon and nitrogen sources and other essential nutrients. Interestingly, however, studies using phenotypic markers to determine the conditions that induce spore germination have suggested that spore germination is induced under conditions that do not support the mitotic cell division cycle [5,6]. Thus, glucose solution without any additional medium components is sufficient to stimulate un-coating, which is an early event in spore germination. In contrast, this solution is not sufficient to induce bud emergence [5]. Under these conditions germination is arrested and the glucose induced-spores rapidly lose viability [6]. The contributions of different components of the medium to changes in molecular processes, such as gene expression, are not known. Characterizing these changes will define the stages at which particular nutrients are needed for this multi-step process.

**Figure 1:**
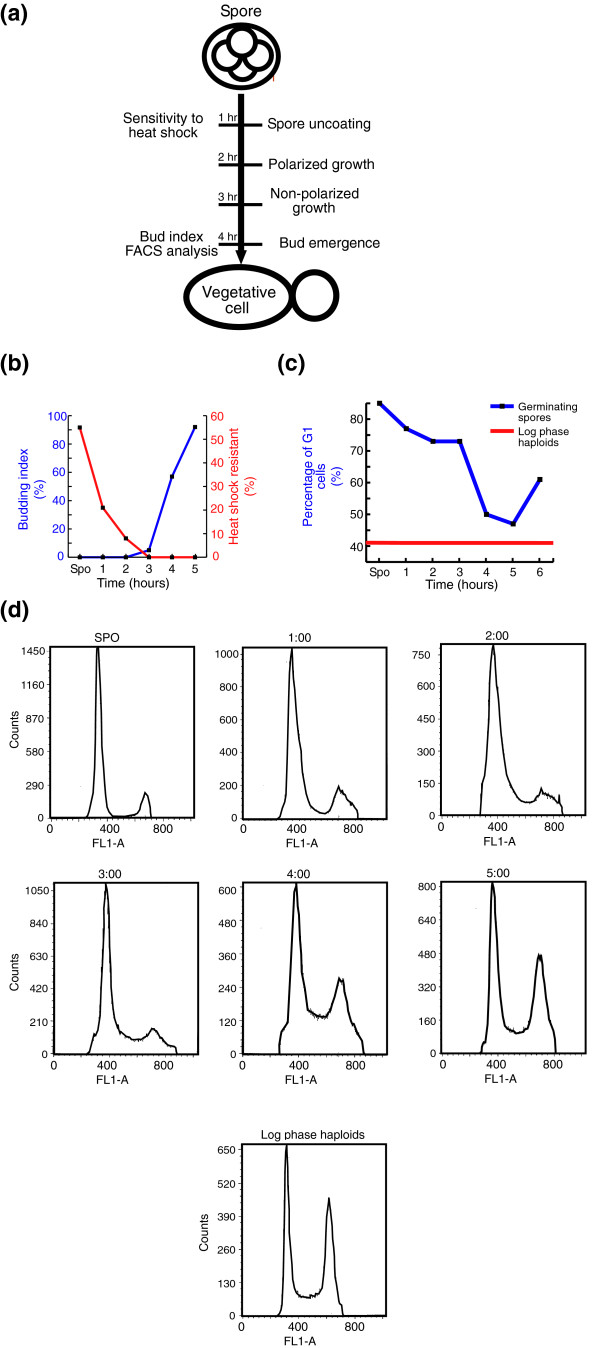
Spore germination in *Saccharomyces cerevisiae *SK1 strain. **(a) **Schematic representation of events known to occur during spore germination in *S. cerevisiae*. See the text for details. **(b) **Budding index and heat shock resistance of germinating spores. Purified SK1 spores were prepared from a diploid strain (DS1) and suspended in YPD medium at 30°C. Samples were taken at the indicated times. Budding index (blue line) was determined by counting 100 cells under the microscope at each time point, using a hemacytometer. For heat shock analysis (red line) aliquots of this germination reaction were removed, diluted and incubated at 55°C for 12 minutes and then plated on solid rich growth medium to determine the number of colony-forming survivors. The percentage of survivors relative to the number of colony forming cells before the heat shock is plotted. **(c,d) **Flow cytometry analysis of germinating spores. Purified spores were prepared from a diploid strain (DS1) and suspended in YPD medium at 30°C. Samples were taken at the indicated times for FACS analysis. Haploid cells were grown in YPD medium to log phase and a sample was taken for FACS analysis. (c) Percentage of G1 cells from all cells is plotted. The red line represents the percentage of G1 cells in log phase haploids. (d) FACS profiles of germinating spores and log phase haploids.

Germination of spores requires a complete change in the state of the cell, involving extensive morphological changes, and changes in metabolism, cellular contents and other physiological properties; it is a multi-step process (Figure 1a). Relatively early, the spore goes through a process of un-coating in which it loses its unique spore wall and becomes more sensitive to different environmental stresses [5]. This stage is followed by phases of polarized, and then non-polarized growth [7]. Eventually, the germinating spores resume DNA replication and budding and enter their first mitotic cell cycle. Most previous studies of spore germination were based on morphological assays carried out relatively late in the process or on assays for specific events (spore un-coating) occurring early in germination. Studying the changes in the global expression profile, in contrast to commonly used assays following specific events in spore germination, provides a comprehensive insight into spore germination and enables us to understand the progression throughout this multi-step process. Changes in gene expression during spore germination were shown to occur practically immediately, observed already 15 minutes after the initiation of spore germination [8]. However, while protein synthesis is required for early stages of spore germination [5], the involvement of changes in gene expression during this transition is not well understood. The genome-wide transcription response of *S. cerevisiae *cells during exit from the stationary phase has been reported recently, revealing rapid and intensive transcription changes during this process [9,10].

Here we report the global changes in gene expression patterns during spore germination. We identified two major stages prior to the first mitotic cell cycle. During the first stage the spores respond only to glucose. Glucose is the principal nutrient triggering spore germination, inducing the germination transcription program. This transcription program is very similar to the general transcription response of yeast cells to glucose, representing resumption of growth and the shift to glucose metabolism. During the second phase of germination the cells are able to sense and respond to components in the environment other than glucose (for example, lack of nitrogen).

Although the main part of the transcription response during the first, early phase of spore germination recapitulates the general response to glucose, detailed analysis enabled us to identify unique aspects of it as well. In contrast to the mitotic cell cycle, growth-related events during germination are not coordinated with nuclear events. We find that regulation of mitotic cell cycle genes, the kinetics of the cyclin Clb2 accumulation and septin dynamics all exhibit unique patterns of regulation.

## Results

### The general transcription program of spores exposed to YPD medium

**Figure 2:**
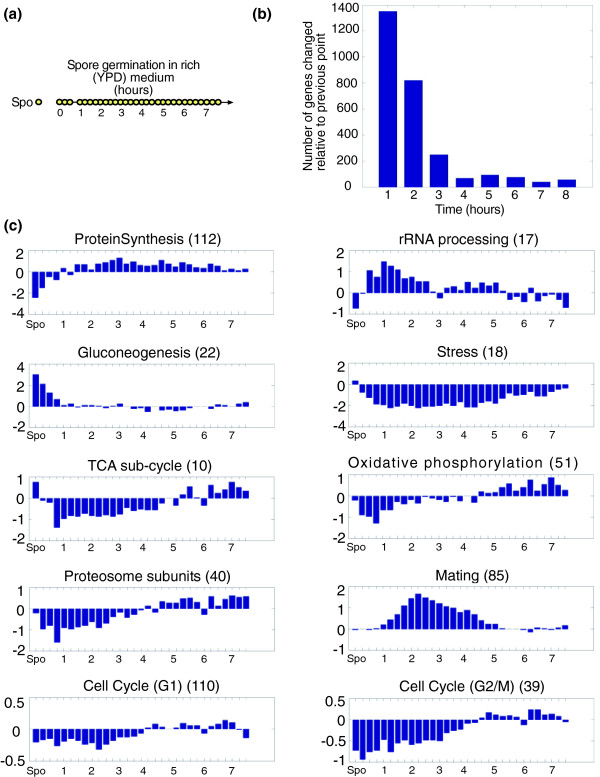
Description of the general transcription response of spores to YPD medium. **(a) **The experimental design. Mature spores (prepared from strain DS1) were incubated in rich medium (YPD) to induce spore germination. Each circle represents a time point at which genome-wide gene expression was monitored; RNA was extracted, labeled and hybridized to a micorarray complementary to all (approximately 6,200) yeast ORFs. The reference RNA was a mixture of RNA from Mata and Matα log phase cells. **(b) **Rapid and intensive changes in gene expression - number of genes whose expression changed at least two-fold compared to the previous time point. To reduce noise, gene expression at each time point was the averaged expression in time points covering one hour, compared to a similar average during the previous hour, except for the first time point, which was compared to gene expression in spores. **(c) **Average expression of genes in specific modules [16] during spore germination. In parentheses is the number of genes in the module. Shown are log2 values of expression relative to expression in vegetative cells. See Additional data file 1 for a complete list of genes that are included in the different modules.

To define the transcription program associated with *S. cerevisiae *spore germination, mature spores of a diploid strain of SK1 genetic background were incubated in rich medium containing glucose (yeast extract/peptone/dextrose (YPD)) to allow spore germination. To follow the pattern of gene expression, samples were taken at high temporal resolution (15 minute intervals for 7.5 hours; Figure 2a). Well-established markers of germination were followed in order to relate changes in gene expression to the different stages of the multi-step germination process. First, we followed the sensitivity of the cells to heat shock. Spores are resistant to heat shock, whereas vegetative cells are sensitive [11,12]. The time when the cells acquire sensitivity to heat shock occurs early in the germination process, within the first one or two hours of transfer to rich medium (Figure 1b). Second, we followed the kinetics of bud emergence. Budding is the most pronounced morphological marker of cycling cells, signifying the transition from G1 to S phase. We observed that buds emerged rather late in the germination process, with only 50% of the spores possessing buds 4 hours after germination was induced (Figure 1b). Early studies of spore germination were limited by poor synchronization of the germinating spores. Spores are usually contaminated with vegetative cells and, therefore, germination is difficult to follow. We therefore used the SK1 strain, which is characterized by its high sporulation rate (>90%). Notably, the kinetics of both bud emergence and sensitivity to heat shock indicate that, under these conditions, SK1 spores germinate with high synchrony relative to previously described spore germination [5,8]. Third, FACS analysis was used to define the beginning of DNA synthesis (Figure 1c,d). In *S. cerevisiae *vegetative cells, bud appearance is synchronized with the initiation of DNA synthesis (G1/S transition). Early studies [13,14] had shown that DNA synthesis is a relatively late event in spore germination, suggesting a correlation between DNA replication and bud emergence during this process. Indeed, DNA synthesis occured four hours after germination was induced, in good correlation with the time of bud emergence (Figure 1b,c).

#### Rapid and intensive changes in gene expression upon transfer of spores to YPD medium

There is some debate whether RNA is synthesized during the early stages of spore germination. Earlier results reported that there was no RNA synthesis during the first hour of germination [14,15]. In contrast, a more recent study showed that RNA synthesis was already active in the first 15 minutes of germination [8]. Consistent with the latter, we observed an extensive change in gene expression at the very early stages of spore germination (Figure 2b). In fact, the expression of about 1,000 genes (out of approximately 6,200) was modified (approximately 550 induced and 480 repressed by at least two-fold) at the first time point examined (after approximately five minutes in YPD medium).

To characterize the transcriptional program of spore germination, we examined groups of genes that are known to be co-regulated [16]. The average expression of genes that are related to specific co-regulated groups is presented in Figure 2c. In addition, we searched for enrichment of specific Gene Ontology (GO) terms amongst the group of genes induced or repressed early in germination (Additional data file 1). This was done using the GO Term Finder tool of the *Saccharomyces *Genome Database [17].

Consistent with the rapid initiation of protein synthesis upon the exposure of spores to growth medium [8,14], the most notable changes in gene expression were the early induction of genes associated with protein translation (rRNA processing and ribosomal proteins) and the repression of genes associated with the presence of a non-optimal carbon source, (for example, gluconeogenesis, TCA cycle, oxidative phosphorylation, proteosome and stress genes; Figure 2c and Additional data file 1).

Genes related to gluconeogenesis and stress are highly expressed in spores and are inhibited immediately as germination starts (Figure 2c and Additional data file 1). The gluconeogenesis pathway is important for long periods of starvation, when glucose must be generated from non-carbohydrate precursors. The changes in the expression of gluconeogenesis and stress genes reflect the shift to glucose metabolism and the release from stress. Similarly, genes that are related to the TCA cycle and to oxidative phosphorylation are expressed in spores, repressed at the beginning of spore germination and induced at a later stage (Figure 2c and Additional data file 1). These results suggest that oxidative phosphorylation and the TCA cycle function in the spores, but are inhibited once glucose is provided and spore germination ensues. Indeed, early studies have shown that spores inherit functional mitochondria, but that germination on glucose is independent of mitochondrial function [13].

Genes coding for components of the proteosome are also expressed in spores and are inhibited as germination begins (Figure 2c). Only little is known about protein degradation and turnover in spores and in resting yeast cells. However, since protein synthesis continues in resting spores [8] while the spores do not grow in mass, it is likely that protein degradation continues as well.

A recent study has suggested that mating may occur among spores within an ascus even before they undergo mitotic divisions [18]. Consistent with that, we observed that genes that are induced during yeast mating are strongly expressed at about two hours following the initiation of germination (Figure 2c). Thus, mating genes are induced long before the germinating spores enter the first cell cycle (at approximately three hours, as detected by the appearance of the first bud; Figure 1b). Using time-lapse microscopy we verified that under our experimental conditions, the germinating spores can also mate before their first buds appear (Additional data file 2).

During spore germination the resting spores re-gain the mitotic cell cycle machinery. We therefore examined the average expression of groups of genes that are co-induced during different stages of the mitotic cell cycle (for example, G1 and G2/M). We expected that genes that are co-induced during the mitotic cell cycle will also be co-induced during this process. However, the change in the average expression of these groups of genes is relatively minor and late (Figure 2c). A modest increase in the average expression of cell cycle genes occurs only after entering the first mitotic cell cycle. More detailed analysis for the involvement of cell cycle genes during spore germination will be described below.

#### Common and unique aspects in the transcription response of spores to glucose

Glucose is a potent and general activator of gene expression. Previous studies have shown that the addition of glucose to cells previously starved of glucose induces rapid and intensive changes in the transcriptional profile of the cells [10,19]. Twenty minutes following the addition of glucose or glucose-rich medium to cells grown on a non-fermentable carbon source or to stationary phase cells, the expression of approximately 2,700 or 2,200 genes, respectively, is modified by more than two-fold. We have noticed that many of the changes in gene expression we observed upon germination are also part of the general response to glucose. This includes the repression of genes involved in gluconeogenesis and oxidative phosphorylation and the increased production of ribosome components and genes involved in protein synthesis [19,20].

**Figure 3:**
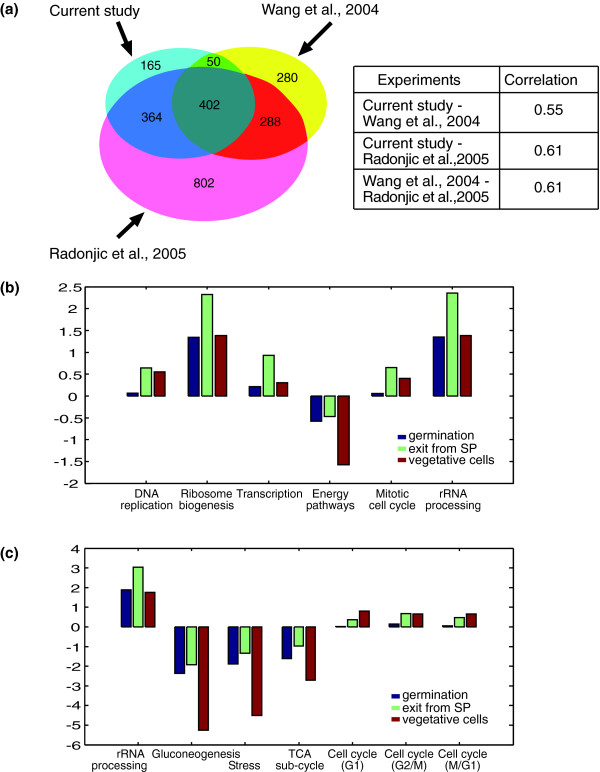
Common and unique aspects in the transcription response of spores to glucose. **(a) **Venn diagram comparing the genes induced during the first hour of spore germination, exit from stationary phase [10] or upon addition of glucose to vegetative cells starved of glucose [19]. A gene was defined as 'induced' if its average expression level during the first hour of the experiment was induced by at least two-fold relative to that before the addition of glucose or glucose-rich media. The area in the Venn diagram is proportional to the number of genes [46]. The correlations between changes in gene expression of all approximately 6,000 genes are indicated for pairs of experiments. Change in gene expression is the average change in expression during the first hour of the experiment relative to gene expression before the addition of glucose or glucose-rich media. **(b,c) **The average expression ratio during the same experiments as in (a) of genes that are related to specific GO terms (b) or in specific modules [16] (c).

To more systematically assess the correlation of the germination transcriptional program we observed with the general response to glucose referred to above, we compared our data to two other published experiments of transcription response following addition of glucose to yeast cells (Figure 3). The first experiment analyzed the transcriptional response following addition of glucose to vegetative cells grown on a non-fermentable carbon source [19], whereas the second considered the exit of cells from stationary phase following addition of glucose-rich medium [10]. Out of 981 genes in our data set induced following the induction of spore germination, 402 were also induced in the other two experiments. Moreover, the overall correlation between the different experiments during the first hour of each experiment is relatively high (Figure 3a), indicating that a major part of the early transcription response observed during spore germination is a component of the general response of cells to glucose.

Thus, a major part of the transcription program we observed correlates with the general response to glucose. To further characterize the similarities and differences in these responses, we focused on specific groups of genes. First, we grouped genes based on their GO classification (Figure 3b). Second, we considered co-expressed gene groups based on the modular composition presented by Ihmels *et al*. [16] (Figure 3c). Indeed, for most gene groups the change in the average expression during spore germination is either similar to, or in-between, the average expression in vegetative cells and upon stationary-phase exit. Notably, however, some exceptions are apparent, with gene groups that behave differently during germination versus the general response to glucose (for example, genes involved in the cell cycle). This germination-specific transcriptional response may reflect specific germination mechanisms, and will be discussed in detail below.

### The contribution of different nutrients to spore germination

Our data and analysis presented above indicate that the transcription response during spore germination principally recapitulates the general response of cells to glucose. This prompted us to examine the contribution of different nutrients to spore germination and to define the stages in germination at which different nutrients are needed. Typically, germination is induced by complete growth medium, either in the form of YPD (rich) or SD (synthetic complete) media. These media contain D-glucose as the carbon source, a nitrogen source and other essential nutrients. We wished to examine the relative contribution of each of the different components to the germination process. An early study examined this issue by following a specific event in spore germination; acquisition of Zymolyase sensitivity was used as an assay for spore un-coating [5]. Glucose was found to be necessary and sufficient to induce sensitivity to Zymolyase, suggesting that glucose alone is sufficient to induce a specific event that occurs early in spore germination. However, as that study followed only one specific event in the process, it could not determine whether glucose induces the full germination program, or is responsible only for this one phenotypic aspect. Indeed, glucose alone is not sufficient for mitotic divisions to take place and, therefore, the induced spores arrest before entering the first cell cycle. As the Zymolyase sensitivity assay examines an early event in spore germination, it cannot be used to follow later progression through the process.

**Figure 4:**
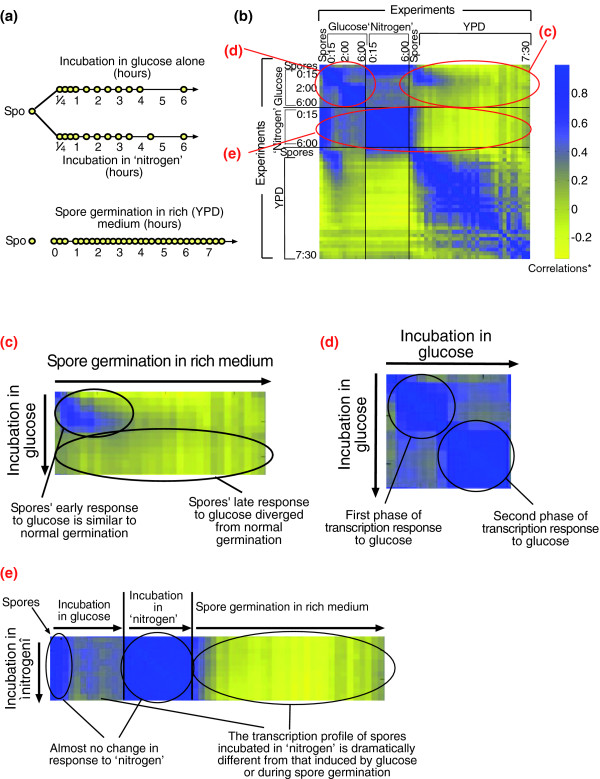
The transcription response of spores to different components of the medium. **(a) **The experimental design. Mature spores (prepared from strain DS1) were incubated in rich medium (YPD) that induces spore germination (Figure 2a) or in either component of the medium (glucose alone or 'nitrogen' - synthetic minimal medium without glucose). Each circle represents a time point at which genome-wide gene expression was monitored; RNA was extracted, labeled and hybridized to micorarray complementary to all (approximately 6,200) yeast ORFs. The reference RNA was a mixture of RNA from Mata and Matα log phase cells. **(b) **The matrix of pairwise correlations describing the similarity between gene expression of all approximately 6,200 genes in the yeast genome for each pair of time points following incubation of spores in glucose, 'nitrogen' or YPD media. Every point represents the correlation between the transcription patterns of two time points. *Correlations are color-coded according to the bar shown. **(c-e)** Enlargements of the parts marked in circles in the correlation matrix presented in (b).

**Figure 5:**
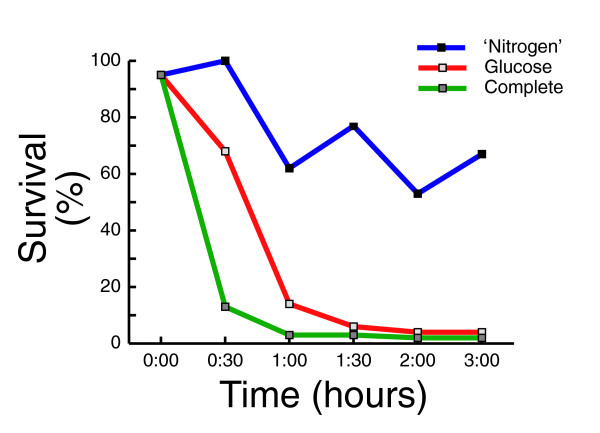
Heat shock resistance of spores incubated with different nutrients. Heat shock analysis was done as described in Figure 1.

We reasoned that studying the changes in gene expression pattern in response to different nutrients could provide a more comprehensive understanding of the aspects of spore germination that are affected by glucose alone. To this end, we separated the complete synthetic medium into its two main nutritional components: the carbon source (2% glucose) and all the remaining ingredients (without glucose, referred to as 'nitrogen source'; see Additional data file 1 for the composition of complete synthetic medium). We incubated mature spores in either component of the medium (glucose or 'nitrogen') and followed, by microarray hybridization, global gene expression for six hours at 15-30 minutes time resolution (Figure 4a). To correlate the observed changes in gene expression to phenotypic progression through germination, we examined also the acquired sensitivity to heat shock (Figure 5). Consistent with previous reports (see above), spores that were incubated in glucose alone acquired sensitivity to heat shock with similar kinetics to spores exposed to complete medium. By contrast, spores exposed to 'nitrogen' (without glucose) remained resistant to heat shock (Figure 5).

#### Glucose is necessary and sufficient to induce an intensive change in the spore's transcription pattern, similar to changes observed during germination in YPD medium

The addition of glucose to mature spores induced a rapid and intensive change in the spores' transcription pattern. In fact, 15 minutes after the addition of glucose, the expression of approximately 1,760 genes was altered over two-fold. This is comparable to the number of genes whose expression varied during actual germination, following the addition of rich medium (YPD). In sharp contrast, 'nitrogen' (without glucose) resulted in a moderate change in the gene expression pattern, with only 362 genes displaying an over two-fold change in expression pattern. Most of the latter (more than 300 genes) were also modified during incubation with glucose alone.

To more systematically compare the germination transcription program with the programs that are elicited by media containing glucose or nitrogen alone, we measured the similarity of the transcriptional responses observed at different time points following the different interventions (addition of YPD, glucose or 'nitrogen' media). Thus, we calculated the Pearson correlation between each pair of arrays, considering all approximately 6,000 yeast genes. The result of this computation is a correlation matrix in which every square represents the correlation between the transcription patterns of two time points (Figure 4b).

The initial transcription response to glucose is highly correlated to and virtually indistinguishable from the response during normal germination in YPD medium (Figure 4c). This response, however, is dramatically different from that induced by 'nitrogen' (Figure 4e). At later times (more than two hours of incubation), the transcription program induced by glucose diverges from that observed in normal germination (Figure 4c). The similarity between the transcription response of spores induced by glucose and during spore germination is clear despite the differences in array analysis (see Materials and methods). Using the same normalization method [21] for all arrays did not affect these results (Additional data file 2). Thus, the changes in transcription program induced by glucose alone can be divided into two phases (Figure 4d). The first phase starts immediately upon the incubation of spores in glucose and continues for 1.5-2 hours, during which there is a gradual change in gene expression. This is followed by a second, relatively static phase that continues for at least four more hours. The gene expression pattern during this second phase is different from those in resting spores, in the first phase of incubation in glucose or in the process of normal spore germination in YPD medium.

**Figure 6:**
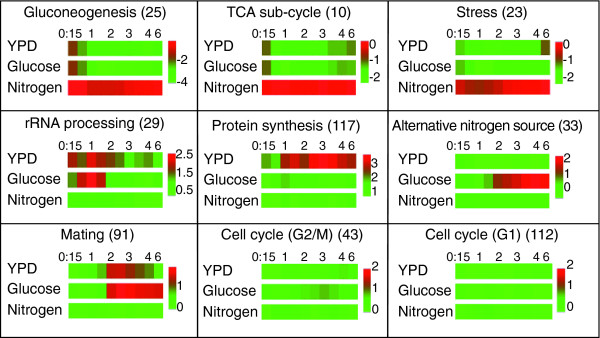
The transcription response of spores to different components of the medium. The average change in expression pattern (relative to spores) of genes in specific modules [16] during spore germination or during spores' incubation in glucose or 'nitrogen'. Expression patterns are shown as log_2 _ratios, and are color-coded for the log_2 _fold change according to the bar shown.

To further characterize the role of glucose in inducing the transcription program of spore germination, we compared the changes in gene expression of specific gene groups upon subjecting mature spores to glucose, 'nitrogen' or YPD media (Figure 6). Genes related to gluconeogenesis, the TCA cycle or stress displayed similar changes in expression pattern during incubation in glucose or in YPD medium. In contrast, the expression of these gene groups did not change during the incubation of spores in 'nitrogen'. Genes related to those processes are repressed during spore germination (in YPD medium) and in response to glucose alone, indicating that glucose alone is sufficient to induce the shift to glucose metabolism and exit from the resting stage of spores.

The immediate increase in expression of rRNA processing genes in response to glucose alone is similar to the induction observed during spore germination, indicating that glucose is sufficient not only to induce exit from the resting state but also for the induction of genes involved in the initiation of growth. Notably, genes coding for ribosomal proteins (protein synthesis genes in Figure 6) are also induced in response to glucose, but this induction is weak relative to their induction by YPD medium. Also, expression of both gene groups is not induced when spores are incubated in 'nitrogen'.

We also examined the response of co-expressed genes that participate in the utilization of alternative nitrogen sources (Figure 6). Genes in this group are repressed by nitrogen and are typically induced when nitrogen is absent. Indeed, no change in the expression of these genes was observed during normal spore germination or when spores were incubated in 'nitrogen' (without glucose). In contrast and not unexpectedly, incubation with glucose alone (without 'nitrogen') resulted in their strong induction. This induction was not immediate but was observed at approximately two hours after the incubation in glucose. Consistent with this, genes involved in translation (rRNA processing and protein synthesis genes in Figure 6), which are induced by glucose with the same initial kinetics as during normal spore germination, were no longer induced at this stage, and were in fact repressed approximately two hours after the addition of glucose, whereas their induction continued in YPD medium. This pattern of expression correlates with the two phases of global gene expression in spores incubated in glucose (Figure 4d). As was discussed earlier, the expression pattern during the first phase following addition of glucose is similar to the expression pattern during normal spore germination. However, the expression pattern during the second phase is distinct.

To further examine the sufficiency of glucose for inducing the later stages of the germination transcription program, we examined the induction of mating genes (Figure 6). During normal germination (in YPD medium), mating genes are induced at approximately two hours and are subsequently repressed. Spores that were incubated in glucose alone, on the other hand, showed mating gene induction at about the same time as spores incubated in YPD medium, but failed to repress these genes. In fact, mating genes remained up-regulated for the full duration of the experiment (six hours). Interestingly, despite this strong induction in mating genes, spores incubated in glucose appeared not to initiate mating and did not form mating projections ('Shmoos'). Thus, it appears that although mating pheromone is being secreted, and the cells respond to it, they can not initiate the morphological changes required for mating. Similar to spore germination in YPD medium, there is relatively little change in the average expression of genes that are co-regulated during the mitotic cell cycle (Figure 6).

#### Glucose induces events related to the cell cycle and advances the time of entering into the cell cycle upon subsequent transfer to rich growth medium

Our results above suggest that glucose is sufficient for initiating the germination process and allowing the cells to enter a growth mode, where they sense the lack of nitrogen; then, at a later stage, the cell cycle arrests but mating events do not take place. This scenario predicts that pre-incubation of spores in glucose would accelerate their subsequent entry into the cell cycle, once nitrogen is also provided. A similar effect of glucose was previously described in cells exiting the stationary phase [6]. If this is not the case, glucose would not initiate the germination program but would induce growth-related events; therefore, it would not accelerate entry into the cell cycle once nitrogen is provided.

**Figure 7:**
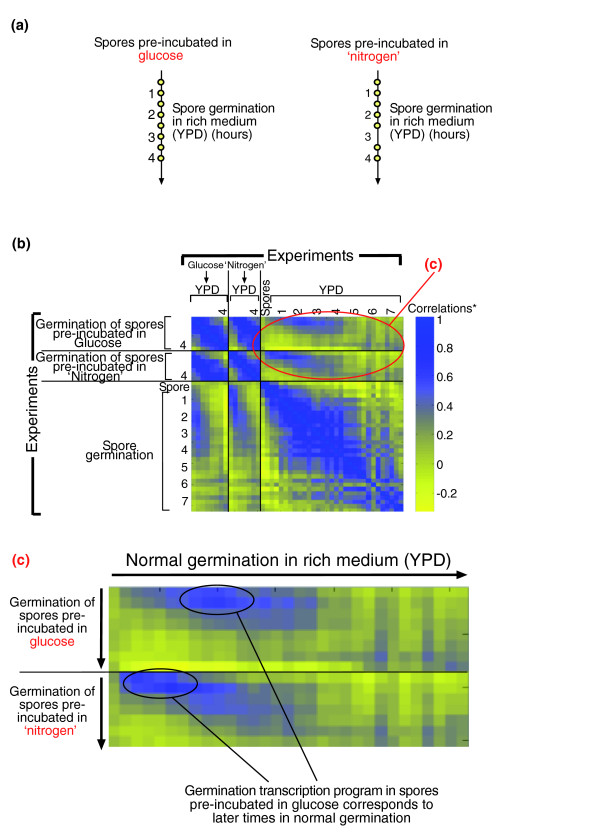
The transcription response to YPD medium of spores pre-incubated in different components of the medium. **(a) **The experimental design. Mature spores (prepared from strain DS1) were incubated in either component of the medium (glucose or 'nitrogen') for two hours and were then transferred to rich medium (YPD) to allow spore germination. Each circle represents a time point at which genome-wide gene expression was monitored; RNA was extracted, labeled and hybridized to micorarray complementary to all (approximately 6,200) yeast ORFs. The reference RNA was a mixture of RNA from Mata and Matα log phase cells. **(b) **The matrix of pairwise correlations (see legend for Figure 4) describing the similarity in gene expression during spore germination (in YPD medium) of spores pre-incubated in glucose or 'nitrogen'. Correlations with normal germination (without pre-incubation, see Figure 2a) are also presented here. *Correlations are color-coded according to the bar shown. **(c) **Enlargements of the parts marked in circles in the correlation matrix presented in (b).

**Figure 8:**
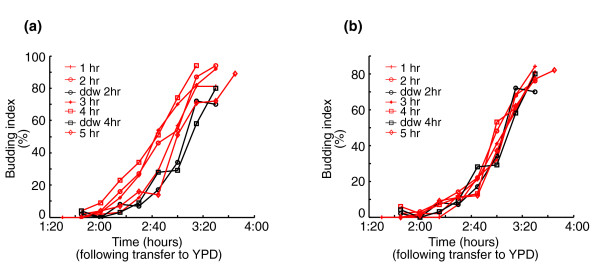
Glucose induces spores to enter the cell cycle. Spores (prepared from the diploid strain DS1) were pre-incubated in **(a) **glucose or **(b) **'nitrogen' for one to five hours and then transferred to rich medium (YPD). Budding index was measured as described before. Time of pre-incubation for each line is in the legend. Black line represents pre-incubation in water.

To examine this prediction, we pre-incubated spores in glucose or nitrogen for two hours and then re-suspended them in rich medium (YPD) to allow spore germination. Samples were taken every 30 minutes for 4 hours for gene expression analysis (Figure 7a). Purified spores were followed by microscopy to characterize the kinetics of bud emergence (Figure 8). As predicted, pre-incubation in glucose shortened the time to bud emergence (Figure 8a), while pre-incubation in 'nitrogen' did not affect budding kinetics (Figure 8b). This advance is also seen by examining the correlation of gene expression profiles in this experiment (Figure 7c). Transfer of both types of pre-incubated spores to YPD medium led to a 'germination-like' profile of gene expression. Despite differences in array analysis (see Materials and methods), the correlation of transcription patterns during germination is high. However, the germination transcription program in spores pre-incubated in glucose corresponds to later time points in normal germination. For example, the gene expression profile of cells that were first incubated with glucose for two hours and then transfered to YPD medium for an additional 30 minutes resembles the gene expression pattern found two hours after the transfer of spores directly to YPD medium (without pre-incubation). In contrast, pre-incubation in nitrogen had no effect on the gene expression program following the transfer to YPD medium. Similar results were obtained when the same normalization method [21] was used for all arrays (Additional data file 2). These results confirm the prediction that glucose not only triggers intensive changes in gene expression in resting spores, but also induces cell cycle events causing spores to be more competent for entering the mitotic cycle.

### Involvement of the mitotic cell cycle machinery in spore germination

#### Genes that are co-regulated during the vegetative cell cycle exhibit a distinct regulatory pattern during germination

Taken together, our results suggest that, to a large extent, the transcriptional program observed during the first two hours of germination is induced by glucose; it is, in fact, very similar to the general program elicited under other conditions, such as addition of glucose to starved cells or the exit of cells from stationary phase. Those results emphasize the principal role of glucose in the initiation of spore germination. However, since the response is so general, it was not clear whether this approach would be useful for identifying or characterizing specific processes occurring during germination. To try to better characterize such processes, we next focused on aspects of the response that appear to be unique to germination, and are different from the general response to glucose observed under other conditions.

**Figure 9:**
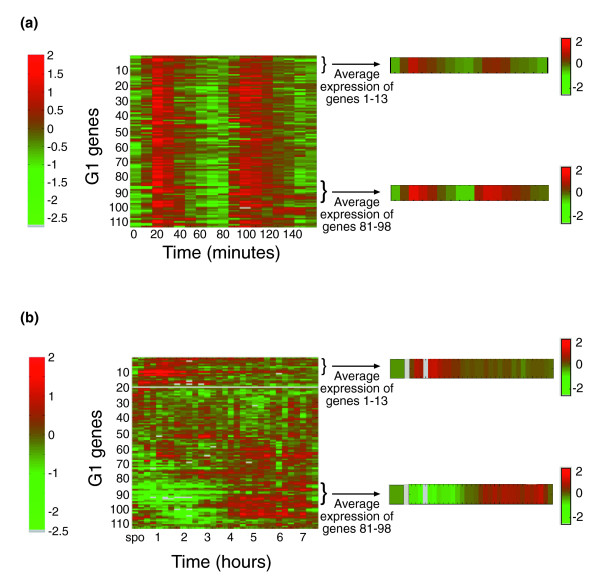
Distinct regulatory pattern during spore germination of genes that are co-regulated during vegetative cell cycle. Expression pattern of genes in G1/S module [16] during **(a) **the mitotic cell cycle in cdc28-13 cells [47] and **(b) **spore germination. Genes were clustered [48] according to their expression during spore germination. Expression patterns are shown as log_2 _ratios, and are color-coded according to the bar shown. Note the difference in time scales between (a) and (b), and that the first cell cycle in germinating spores (b) starts relatively late, after approximately four hours (Figure 1).

We focused first on a group of 112 genes that are co-induced during the G1/S transition of the mitotic cycle, the G1/S module of Ihmels *et al*. [16] (Figure 9a). It is believed that resting cells enter the mitotic cell cycle through the G1 phase [22]. We thus expected this group of genes to be co-induced before entering the first cell cycle. In contrast to the coherent co-expression of these genes during the mitotic cell cycle, their behavior in our experiment was dramatically different. Clustering of these genes according to their expression during spore germination revealed that they are not, in fact, co-expressed during spore germination (Figure 9b). We identified two distinctly co-regulated groups of genes, whereas a large number of genes (80 out of 110 'G1 genes') were not co-regulated in any distinct pattern. Genes in the first cluster (genes 1-13; see Table 1 for gene list) were induced early, less than one hour after the initiation of germination. This early induction precedes the induction of mating genes and was significantly earlier than bud emergence. In contrast, genes in the second cluster (genes 81-98; see Table 1 for gene list) were repressed early in germination and were induced only later, approximately four hours after the addition of YPD medium, corresponding to the timing of bud emergence. Thus, whereas during the normal cell cycle these genes appear as a single co-regulated group [16], our results indicate that they have different regulation patterns during spore germination.

**Table 1:** G1/S genes that are co-regulated during spore germination

Gene	Description
**Genes expressed early in germination (genes 1-13 in Figure 10)**	
*YBL009W*	Protein of unknown function
***RDH54***	Protein required for mitotic diploid-specific recombination and repair and for meiosis
***HCM1***	Dosage-dependent suppressor of cmd1 and member of the forkhead family of DNA-binding proteins
***SHO1***	Osmosensor in the HOG1 MAP kinase, high-osmolarity signal transduction pathway, has an SH3 domain
***YGR151C***	Protein of unknown function
***RSR1***	GTP-binding protein involved in bud site selection, member of the ras family in the ras superfamily
*YKL089W*	Centromere protein required for normal chromosome segregation and spindle integrity
***SPH1***	Protein involved in polarized growth, with roles in shmoo formation and bud site selection
***OGG1***	DNA glycosylase, excises 7,8-dihydro-8-oxoguanine (8-OxoG) and formamidopyrimidine (Fapy) residues from DNA
***BNI5***	Protein of unknown function, localizes to the mother-bud neck
***BNI4***	Protein that may be involved in linking chitin synthase III to septins of the neck filaments
***EXO1***	Double-stranded DNA 5'→3' exonuclease, involved in mismatch repair and recombination
***BBP1***	Protein of the spindle pole body that binds to Bfr1p
**Genes expressed late in germination (genes 81-98 in Figure 10)**	
***RFA1***	DNA replication factor A, 69K subunit, binds single-stranded DNA
***HTA2***	Histone H2A, nearly identical to Hta1p
***POL30***	Proliferating cell nuclear antigen (PCNA), required for DNA synthesis and DNA repair
***MCD1***	Cohesin, protein required for mitotic chromatid cohesion and chromosome condensation
***HTA1***	Histone H2A, nearly identical to Hta2p
***RNR1***	Ribonucleotide reductase (ribonucleoside-diphosphate reductase) large subunit, converts ribonucleoside diphosphate to deoxyribonucleoside diphosphate
***RFA3***	DNA replication factor A, 13K subunit
*YLR049C*	Protein of unknown function
***TOS4***	Protein of unknown function
***YOX1***	Protein with a homeodomain that binds tRNA-Leu gene
***CTF18***	Protein required for accurate chromosome transmission in mitosis and maintenance of normal telomere length homolog of Rfc1p, Rfc2p, Rfc3p, Rfc4p, and Rfc5p
***HHF2***	Histone H4, identical to Hhf1p
***RFA2***	DNA replication factor A, 36K subunit phosphorylated at the G1/S transition and dephosphorylated at mitosis
*YNR009W*	Protein of unknown function
***CSI2***	Protein involved in chitin synthesis
***MSH2***	Component with Msh3p and Msh6p of DNA mismatch binding factor, involved in repair of single base mismatches and short insertions/deletions
***CDC21***	Thymidylate synthase, catalyzes the reductive methylation of dUMP by 5,10-methylene-5,6,7,8-tetrahydrofolate to produce dTMP and 7,8-dihydrofolate
***HHO1***	Histone H1

To further analyze the two distinctly expressed G1 groups of genes, we examined their composition (Additional data file 1). There is a clear difference between the two groups. The early group (genes 1-13 in Figure 9b) is enriched in genes that are related to cytoskeleton organization, cytokinesis and polar budding. In contrast, the late group (genes 81-98 in Figure 9b) is enriched in genes related to DNA replication and DNA metabolism (Additional data file 1). The induction of these genes occurs in parallel to bud appearance (Figure 1b) and DNA synthesis (Figure 1c). The unique expression pattern of G1 genes suggests that components of the mitotic cell cycle machinery are involved in spore germination, but this involvement has a unique pattern, which is germination-specific. Not much is known about the involvement of the basic cell cycle machinery in spore germination. We used two cell cycle markers, the septin Cdc10 and the cyclin Clb2, to follow the timing and involvement of the mitotic cell cycle machinery in germination.

#### Cdc10 protein dynamics throughout spore germination

**Figure 10:**
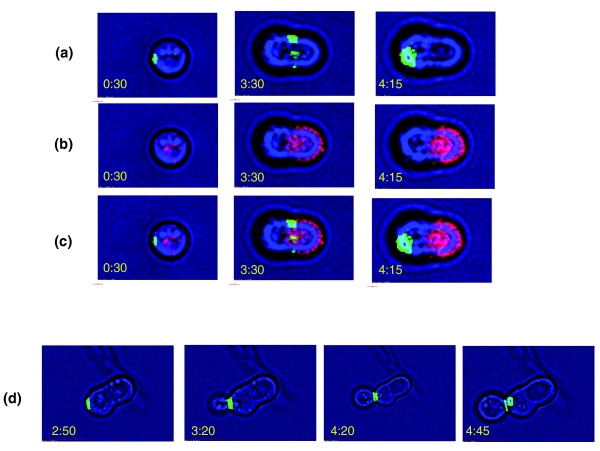
Cdc10-GFP protein dynamics throughout spore germination. Purified spores containing GFP-tagged Cdc10 (prepared from strain DS38) were plated and synthetic minimal medium was added to allow spore germination. Time lapse microscopy was carried out using a Deltavision RT microscope system with OAI Scan command and the results deconvolved. **(a-c) **Spore germination before the appearance of the first bud. Images were monitored at 60× magnification using (a) FITC (excitation 490 and emission 526) filter, (b) RD-TR-Cy3 (excitation 555 and emission 617) filter and (c) merged pictures of (a) and (b). **(d) **The first mitotic cell cycle was monitored at 100× magnification using a FITC filter. In yellow is the time following the addition of synthetic minimal medium in hours and minutes.

In the mitotic cell cycle, rearrangement of septins directs bud emergence, bud growth and cytokinesis [23,24]. Septins are involved in different aspects of morphogenesis, such as selection of cell polarity and the switch from polar to isotropic bud growth. To ensure synchronization between cortical and nuclear events, septin dynamics are regulated by the mitotic cell cycle controls [24]. Septins, therefore, are useful markers for progress through the cell cycle. Cells containing a green fluorescent protein (GFP) labeled septin (Cdc10-GFP) were used to follow septin dynamics throughout spore germination. Importantly, the Cdc10-GFP tagged protein forms typical septin structures throughout the mitotic cell cycle (Figure 10d) and cells expressing this tagged protein did not show any detectable change in overall phenotype.

We observed a clear signal from Cdc10-GFP in spores (Additional data file 2; Figure 10a, 0:30). The Cdc10-GFP fluorescence signal appeared as a concentrated signal at one edge of the spore. Notably, this localized position of Cdc10 marked the site of polarized growth during germination. Following the induction of spore germination, polarized growth is seen preferentially at the edge marked with Cdc10-GFP. At a later time, the bud emerges at this end. This finding shows that resting spores contain signals marking the direction of growth. Although the diploid cells used for sporulation are heterozygous *CDC10-GFP/CDC10 *and, therefore, only half the spores contain the gene *CDC10-GFP*, the tagged protein appears in almost all spores (data not shown). However, the signal disappears in approximately half of the germinating cells and becomes stronger in the other half. This result suggests that the Cdc10 protein in mature spores originated before spore formation, not later than the meiotic divisions. In addition, we have noticed that while using the RD-TR-Cy3 filter (excitation at 555 nm and emission at 617 nm), the spores show red fluorescence at this wavelength (Figure 10b, 0:30). This auto-fluorescence was further used to distinguish between different domains of the germinating spores (next section).

As described above, Cdc10-GFP was easily detected in spores, marking their growth site. Later, the signal disappears and, just before bud emergence, it re-appears, being localized to the pre-bud site and then to the bud neck (Figure 10a, 4:15, and 10d). The latter kinetics are similar to those observed during budding in vegetative cells. However, after spore germination begins, but before buds start to appear, Cdc10 can not be detected at the site of growth. It can be detected, although at a much lower intensity, as a band separating the two unequal halves of the germinating spore (Figure 10a, 3:30). The disassembly and re-assembly of Cdc10 may indicate a possible role for Cdc10. A clue to Cdc10's role during this early stage of germination could be obtained from another interesting phenomenon that we observed during our work and mentioned at the end of the previous section. While using the RD-TR-Cy3 filter on growing spores, we noticed that as soon as the spores begin to grow unidirectionally (by the 'polar growth' phase), there is a clear distinction between the two parts of each spore. At this stage the auto-fluorescence of the spore (see previous section) is becoming stronger but only in the non-growing half of the germinating spore, while the growing half is not fluorescent (Figure 10b). We used this label to identify the border between the growing and non-growing domains ('halves') of the spore. By merging RD and GFP signals it can be seen that Cdc10 is localized to the border that separates the growing and non-growing parts of the spore (Figure 10c, 3:30).

Our results suggest the involvement of the mitotic septin (Cdc10) in spore germination, before the buds start to emerge. However, whereas during the mitotic cell cycle septin regulation is highly coordinated with other cell cycle events [24], our results suggest that, during spore germination, Cdc10 undergoes a different pattern of regulation.

#### Accumulation of Clb2 protein during spore germination

**Figure 11:**
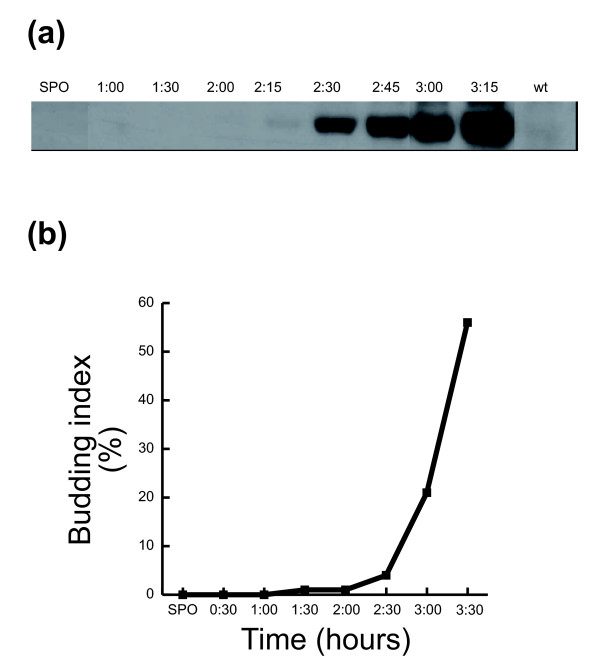
Early accumulation of Clb2 protein. Purified spores containing HA-tagged Clb2 (prepared from strain DS35) were transferred to rich medium to allow spore germination. At the indicated time, samples were taken for **(a) **western blot analysis and **(b) **budding index analysis.

Cyclins play a prominent role in directing cell cycle oscillations. During the mitotic cell cycle the level of transcripts of most cyclins oscillates, as do the levels of the proteins themselves. Hence, characterizing cyclin levels provides a good indication of the cell cycle stage. We therefore decided to characterize the levels of Clb2 protein during spore germination. Clb2 is the principal cyclin in the mitotic cell cycle. It is absent from spores and the gene is not expressed during meiosis [3]. We used cells containing hemagglutinin (HA) tagged Clb2 to follow the protein levels during spore germination (Figure 11a). The protein first becomes apparent 2 hours and 15 minutes after germination begins (Figure 11a), coincidental with the second phase of germination (Figure 4d). Then, Clb2 protein accumulates, reaching high levels at 3 hours and 15 minutes. Interestingly, the mRNA level of Cdh1, which is involved in Clb2 proteolysis during the mitotic cell cycle, is high in spores (it correlates with low levels of Clb2) and is repressed during the first two hours of spore germination, in correlation with Clb2 accumulation.

Clb2 protein accumulation as well as associated H1 kinase activity is known to begin at late S or early G2 phase of the mitotic cell cycle and reach a maximal level at the time of mitosis, followed by a reduced level [3]. Comparing the relative kinetics of Clb2 accumulation and budding during spore germination (Figure 11) and throughout the mitotic cell cycle [3] suggests that, during spore germination, Clb2 is induced earlier than during the mitotic cell cycle and may even precede the appearance of buds and DNA synthesis. Clb2 protein accumulation begins at 2 hours and 15 minutes, coincident with the initiation of budding in less than 4% of the germinating spores. Thus, the timing of Clb2 appearance in relation to DNA replication is different in spore germination from that found in the mitotic cell cycle.

## Discussion

**Figure 12:**
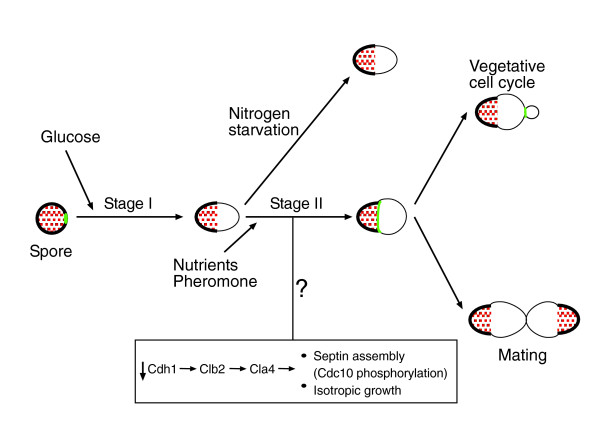
Events occurring during spore germination. Our results suggest that spore germination can be divided into two major stages. Stage I is induced by glucose alone whereas for the transition to stage II other nutrients are necessary. Mitotic cell cycle machinery is involved in spore germination but, in contrast to the cell cycle, growth related events are regulated separately from nuclear events. A model for activation of cell cycle events that occur during stage II of germination is suggested at the lower part of the figure. See Discussion for more details. Septin (Cdc10) is depicted in green. Auto-fluorescence of the spore is depicted in red.

Spore germination in *S. cerevisiae *is a multi-step process in which the resting haploid spores resume growth and enter the mitotic cell cycle. Hence, spore germination is not only highly important for the life cycle of the organism but it also provides a model for studying the mechanisms underlying exit from quiescence and entry into the cell cycle. Still, little is known about this process. Most early studies of spore germination used assays that measure unique phenotypic events [5] and could not follow the progression throughout the process. In this study we examined the global changes in gene expression during spore germination and followed cell-cycle and germination markers. A model for a temporal scheme of events summarizing this work is presented in Figure 12.

Spore germination is induced upon addition of glucose-rich medium to spores. Gene expression profiles during normal germination change gradually throughout the whole process. However, genome-wide expression analysis of spores incubated in glucose alone suggests that this process can be divided into two distinct stages (Figure 4). The first stage starts immediately upon induction of germination and continues for 1.5-2 hours. This is followed by a second stage that continues until the germinating spores enter the first mitotic cell cycle.

The transcription program during the first stage of germination is highly similar to the general transcription response of yeast cells to glucose. Similar to the general response to glucose, we observed a rapid and intensive change in gene expression pattern following the induction of spore germination (Figure 2). The extent to which gene expression is modulated during spore germination was not appreciated before. In fact, it was generally assumed that new transcription does not take place during the first stages of spore germination. A recent study [8] was the first to report that transcription of specific RNAs occurs during the first hour of spore germination. Here, we significantly extended those results, by showing that early germination involves a large-scale change in the gene expression pattern.

The transcription program during early germination closely resembles the rapid and extensive changes in gene expression observed upon exit from stationary phase [9,10]. Our systematic comparisons of spore germination to processes occurring upon addition of glucose to cells that are starved of glucose [10,19] have revealed that the majority of changes in gene expression pattern during early spore germination are part of the general response to glucose (Figure 3). The common changes in gene expression pattern reflect the shift to glucose metabolism and the initiation of growth that occurs in the germinating spores during this stage. Moreover, glucose alone is necessary and sufficient to induce a transcription pattern that is almost indistinguishable from that found during the first stage (two hours) of germination in YPD medium (Figures 4 and 6). Indeed, in the presence of glucose the spores lose their unique characteristics [5,6] (Figure 5) and become more competent to enter the mitotic cell cycle (Figures 7 and 8). In contrast, we observed that when glucose is absent from the medium ('nitrogen' medium) there is almost no change in gene expression pattern relative to the expression pattern found in resting spores (Figures 4 and 6).

Our results indicate that only during the second phase of germination (starting about two hours after germination was initiated) are the cells able to respond to the environment (Figure 12). At this stage in normal germination, mating genes are induced (Figure 2), indicating that the spores have gained the ability to respond to mating pheromone. In addition, it appears that only during this stage are the cells able to respond to lack of nitrogen, as indicated by the increase in the expression of genes responsive to nitrogen starvation at this stage (Figure 6). Consequently, when nitrogen is absent from the medium, cell growth is arrested at the onset of this second stage, mating events are prevented and the spores enter a distinct developmental phase. Not much is known about processes occurring at this phase during normal germination. However, studying the morphological changes during germination has revealed that this stage is characterized by de-polarized growth of the spore [7].

The mechanisms underlying spores' unresponsiveness to different environmental cues have to be further investigated. A spore might be isolated from its environment until the beginning of stage II. It may not utilize all mechanisms that enable cells to sense and respond to different components of the medium. This can also explain the kinetics of mating genes' expression. However, it is also possible that the carbon source is the only limiting factor for the beginning of spore germination. Thus, only during the second stage are other components needed and the spore responds to their absence. In addition, further characterization of spores' unresponsiveness is needed. Gene expression responses of the germinating spores to different environmental stresses (for example, heat shock) have yet to be determined.

The general understanding is that in *S. cerevisiae*, growth and cell division are coordinated during the G1 phase of the cell cycle, such that all growth requirements are met before the cell commits to a new cell cycle and division (through START). Accordingly, it is usually assumed that increases in doubling time reflect prolonged G1 phase. A recent study [25] contrasted this view, suggesting that different nutrient limitations (for example, nitrogen and glucose limitations) differentially affect cell cycle progression. Thus, it was shown that under nitrogen limitation, non-G1 phases expand almost as much as G1 [25]. Our results suggest that during spore germination, sensing of different nutrients (glucose and nitrogen) occur at different stages; glucose is sensed by the resting spores whereas nitrogen is sensed only at the following stage of germination. Notably, nitrogen sensing still occurs approximately two hours before the germinating spores start the first mitotic cell cycle and go through START (Figure 12).

Taken together, our genome-wide analysis has enabled us to establish the principal role of glucose in triggering spore germination. We have seen that, to a large extent, the transcriptional program observed during the first two hours of germination is induced by glucose, and is in fact highly similar to the general program induced by glucose addition to vegetative cells starved of glucose. Detailed characterization of the global transcription pattern has enabled us to define the stages in the process at which other nutrients are needed and can be sensed.

A major goal of this work was to characterize cell-cycle related processes that are specific to spore germination. Although the major part of the transcription response program during the early phase of spore germination recapitulated the general response to glucose, we have identified a unique transcription profile of some genes that are related to the cell cycle and to DNA replication (Figure 3). Detailed analysis of a group of genes that are co-induced during G1/S phase of the mitotic cell cycle has revealed unique regulation for these genes during spore germination (Figure 9). In particular, two sub-groups of genes that differed in their expression patterns during germination were found to be associated with distinct functions (as revealed by analyzing their associated GO categories). The first group was induced early, during phase I of spore germination (Figure 12) and was enriched in genes related to cytoskeleton organization and polar budding (Additional data file 1). In contrast, the second group, which was repressed early in germination but was induced approximately four hours after initiation of germination, was enriched by genes related to DNA replication (Additional data file 1). The unique regulation pattern of G1/S genes suggests that these genes are involved in spore germination. The kinetics of their expression are consistent with the fact that those processes occur at different times during spore germination than in the mitotic cell cycle (in the latter budding and DNA replication occur concomitantly). Therefore, the two sub-groups of genes are separable from each other and from the rest of G1/S genes. Indeed, a previous study has shown that the initiation of spore germination is closely followed by a phase of polar growth [7], occurring during phase I of spore germination (Figure 12), while DNA replication is known to begin only at a later stage [13,14] (Figure 1c), in parallel with induction of the late sub-group.

How can the cells achieve this distinct regulatory pattern? During the extensive transcriptional changes that occur upon addition of glucose to vegetative cells grown on a non-fermentable carbon source [19], genes in the G1/S module [16] are induced as well. In vegetative cells, most of the transcriptional effects of glucose addition are regulated redundantly by a Ras-dependent pathway and by one or more Ras-independent pathways [19]. However, DNA replication genes are highly enriched by a small group of genes that were found to be regulated only by a Ras-independent pathway and were not affected by activation of Ras signaling [19]. Indeed, although both our sub-groups of the G1/S module are induced by glucose, genes that are expressed early in spore germination (the first group) are induced by the Ras-dependent pathway [19], whereas genes that are expressed late in spore germination and are enriched in genes related to DNA replication (the second group) are induced only by the Ras-independent pathway [19]. Interestingly, the Ras signal transduction pathway is a key regulator of spore germination and is necessary for early events [5]. Therefore, our results (Figure 9 and Additional data file 1) suggest that during spore germination, the signaling pathways mediating glucose response are not redundant; the Ras-dependent pathway is activated early in the process, mediating the immediate transcription response, whereas at least one Ras-independent pathway is not activated at this stage. DNA replication genes that are not affected by the Ras signaling pathway are, therefore, induced at a later time, following the beginning of the first cell cycle.

The involvement of the mitotic cell cycle machinery in spore germination was suggested by our gene expression profiling (Figure 9). We then examined it more directly by using two cell cycle markers, the septin Cdc10 and the cyclin Clb2. During the mitotic life cycle, cells that exhibit axial budding utilize a cytokinesis tag from the preceding cell cycle that directs the formation of the new bud to an adjacent site [26]. Septin proteins have been proposed as components of this cortical tag. The septins are required for bud site selection, presumably by acting as a scaffold to direct localization of signal molecules to the potential bud sites [27]. Interestingly, we have seen that Cdc10 is tightly localized already in the resting spores, marking the direction of polarized growth during germination (Figure 10 and Additional data file 1). This result suggests a role for septins in selecting the direction of growth also during spore germination. Thus, this molecular tag indicates a cellular connection between the meiotic process, spore germination and the following (first) mitotic cell cycle.

Septins were proposed to maintain cell polarity during the mitotic cell cycle. By specifying a boundary between cortical domains, septins function to prevent lateral diffusion of membrane-associated proteins [28]. In particular, septins were found to form a boundary during the isotropic bud growth phase, between the active bud surface and the relatively quiescent surface of the mother cell [29]. Our results (Figure 10) suggest a similar role for septins in phase II of germination (Figure 12), following induction of spore germination but before bud appearance and entry into the mitotic cell cycle. Cdc10 dynamics suggest that the germinating spores' polarity is maintained by forming a cortical barrier during the isotropic growth phase between the growing and non-growing parts of the spore. During the mitotic cell cycle, septin regulation is highly coordinated with other cell cycle events to maintain synchronization between cortical and nuclear events [24]. In contrast, our results indicate that in spore germination, Cdc10 dynamics are separated from typical cell cycle events.

We also followed the accumulation of the cyclin Clb2 during spore germination (Figure 11). Also here, a unique pattern of regulation was identified. During the vegetative cell cycle, accumulation of Clb2 starts in late S phase and continues up to mitosis [3]. In spore germination, Clb2 is induced earlier, maybe even during phase II of this process (Figure 12), before the initiation of the first mitotic cycle. This result suggests involvement of Clb2 in spore germination. During the mitotic cell cycle, Clb2 has a key role in nuclear division [30,31] and in the transition from polar to isotropic growth, occurring also during the M phase of the mitotic cell cycle [32]. Recently, it was shown that during phase II the germinating spores undergo a transition from polar to isotropic growth that is regulated by a number of factors also implicated in mitotic bud morphogenesis [7]. Therefore, during germination Clb2 starts to accumulate at the same time as the switch to isotropic growth, suggesting Clb2 involvement in this transition. However, while nuclear division and isotropic growth occur at the same time in the mitotic cell cycle, during germination the switch to isotropic growth precedes the first mitotic cycle and nuclear division. Hence, Clb2 activity during spore germination is separated from other, nuclear, cell cycle events. Our results suggest that Clb2 is involved in the switch to isotropic growth during spore germination, but its direct involvement remains to be shown. A previous study has failed to show the involvement of cyclin-dependent kinase Cdc28 in spore germination [5]. However, that study used an assay for an early event in spore germination only (Zymolyase sensitivity), and could not detect a requirement for these proteins later in the process.

The accumulation of Clb2 before the first mitotic cell cycle contradicts its inhibitory affect on the ability of cells to construct an incipient bud site during G1 [33]. The germinating spores can bud, despite high levels of Clb2 (Figure 11). One possible explanation for this is that Clb2 is not active in the germinating cells. Recently, the highly robust nature of this system was demonstrated, as constitutive expression of Clb2 did not reduce viability of the cells [34]. This raises the possibility that also in spore germination, Clb2 activity is not high enough to inhibit bud site assembly. The involvement of Clb2 protein during phase II of spore germination has yet to be established.

Cdh1 serves as an activator of the APC and mediates ubiquitin-dependent protein degradation of the mitotic cyclin Clb2 [35]. Interestingly, we have seen that *CDH1 *mRNA is high in spores and is repressed before the end of phase I of germination in correlation with Clb2 accumulation. During the mitotic cell cycle, Clb2 is known to induce hyper-phosphorylation of Cla4 [36], which was found to be involved in the isotropic growth occurring during phase II of spore germination [7]. Cla4 is also known to directly phosphorylate septins Cdc3 and Cdc10 and to be involved in septin ring assembly during the mitotic cell cycle [37]. This suggests that in spores and during stage I of germination, Cdh1 is involved in Clb2 degradation (Figure 12). *CDH1 *repression (during stage II) induces Clb2 accumulation. Clb2 then induces Cla4p, which is involved in the isotropic growth phase and in septin assembly at the border of the spore (Figure 10). Further experiments are required to examine this hypothesis.

Taken together, genome-wide analysis has enabled us to identify unique aspects of spore germination, suggesting an involvement of the mitotic cycle machinery in this process. The main take-home message is that, in contrast to the mitotic cell cycle, growth related events and nuclear events are regulated differently during spore germination

In conclusion, our study suggests that spore germination can be divided into two major stages. In our model (Figure 12) the transition between stages I and II involves major changes in the germinating spores. During this transition germinating spores become sensitive to the environment, starting to sense mating pheromones and nutrients and maybe also other external signals. The spore then switches to an isotropic growth mode, septins are assembled at the border between the growing and non-growing parts of the spore and the cyclin Clb2 starts to accumulate. All these processes occur at the same time, suggesting that the spores undergo a fundamental change during this transition.

## Materials and methods

### Strains of *Saccharomyces cerevisiae*

All strains are of SK1 genetic background and their genotypes are listed in Additional data file 1. DS6, a strain containing CDC10-GFP, was constructed by one-step PCR-based replacement method. PCR was performed using a strain containing GFP-labeled Cdc10 (from the yeast GFP clone collection, purchased from Invitrogen) as a template and the primers CDC10-F and CDC10-R (Additional data file 1). The haploid strain, D277, was transformed with the PCR product. Strains were selected on synthetic minimal plates lacking histidine. Integration to the correct site was verified by PCR using CDC10-CHK and the universal reverse primers (Additional data file 1).

DS28 and DS29, strains containing *CLB2*-3HA, were constructed by oligonucleotide-directed homologous recombination system [38] as described before [39]. PCR was performed using pFA6a-3HA-kanMX6 plasmid [38] and primers CLB2-R and CLB2-F (Additional data file 1). The haploid strains, NKY1059 and NKY561, were transformed with the PCR product and G418-resistant transformants were selected on YPD+G418 plates. Integration of the cassette to the correct site was verified by PCR using primers CLB2-CHK and U-CHK (Additional data file 1). All transformations were done using the lithium acetate method [40].

### Sporulation and germination conditions

Cells were grown to saturation in YPDx2 at 30°C. The cells were then washed in sterile water and plated on sporulation medium (SPO; Additional data file 1) plates (140 mm Petri dishes) at 30°C. Three- to five-day-old asci were harvested in sterile water using the handle of a Drigalski Spatula. To initiate spore germination, asci were suspended at approximately 1.5 × 10^7 ^cells/ml in glucose containing medium (either YPD or synthetic minimal; Additional data file 1) at 30°C with shaking. To examine the contribution of different nutrients to spore germination, 3- to 5-day-old asci were suspended, at approximately 1 × 10^7 ^cells/ml, in either glucose (2% glucose) or 'nitrogen' (synthetic minimal medium without glucose; see Additional data file 1 for the composition of synthetic minimal medium).

Intact asci were used for gene expression experiments presented here, while for budding index, heat shock analysis, time-lapse microscopy and western blot analysis we used purified spores. Spore purification was performed as described previously [5] with minor modifications. A pilot experiment has shown a high correlation between changes in gene expression profiles in germinating asci versus purified spores (data not shown). Time-lapse microscopy was done using a Deltavision RT microscope system (Applied Precision Inc., Issaquah, WA, USA).

### Heat shock analysis, budding index and flow cytometry analysis

Heat shock analysis was performed by incubating cells at 55°C for 12 minutes and then plating them on solid rich growth medium (YPD). The number of survivors was determined as the percentage of colony-forming survivors after heat shock, relative to the colony forming cells before the heat shock. Budding index was determined by counting 100 cells under the microscope at each time point, using a hemacytometer. DNA content of cells was analyzed by flow cytometry (FACS). Cells were fixed in 70% ethanol, treated with RNaseA and proteinase K and then stained with SYBR green (200 μl (1:1,000) SYBR green/10^7 ^cells, for 1 hour in the dark) and sonicated, before being analyzed in a FACSCalibur analyzer (Becton-Dickinson, San Jose, CA USA).

### Preparation of yeast protein extracts and western blot analysis

Protein extracts were prepared from trichloroacetic acid-treated cells and protein concentrations were determined essentially as described previously [41,42]. For western blot analysis, equal amounts of proteins were separated on by 10% SDS-PAGE, blotted onto nitrocellulose membranes (0.45 μm), reacted with monoclonal antibody (12CA5) directed against the HA epitope at a concentration of 1:6,000, and visualized by enhanced chemiluminescence.

### RNA extraction and labeling

For RNA extractions, samples were collected and spun at 2,000 rpm for 7 minutes at room temperature, flash frozen in liquid nitrogen and kept at -80°C until RNA extraction. Yields of RNA extractions from spores are relatively low and increase during spore germination. Therefore, sample size to yield more than 20 μg RNA was determined by a preliminary experiment. Total RNA was extracted using the RNeasy Midi Kit (Qiagen, Valencia, CA, USA) and reverse transcribed using M-MLV reverse transcriptase RNase H Minus (Promega, Madison, WI, USA). cDNA products were labeled with Cy3 and Cy5 by the indirect amino-allyl method [43], with minor modifications. Dye incorporation was measured using a spectrophotometer. Spores for gene expression experiments were prepared from a diploid SK1 strain (DS1). Reference RNA for all microarrays in this work was a mixture of RNA from MATa (NKY1059) and MATα (NKY561) vegetative haploid cells, grown separately to log phase.

### Microarray hybridization scanning and quantification

For each hybridization, cDNA samples were labeled with Cy3 and Cy5 and combined with blockers: 5 μg herring sperm (Promega), 5 μg tRNA (Gibco) and 17.5 μg poly(A) (poly(A) oligonucleotides were synthesized at mixed lengths of 40, 50, and 60 adenine residues). The labeled cDNAs were concentrated to 40 μl using Microcon (Millipore, Bedford, MA, USA) and 40 μl of 2× hybridization solution (10× SSC, 50% formamide, 0.2% SDS) was added. Microarrays containing all yeast open reading frames (ORFs) were pre-hybridized by incubation in a solution containing 1% bovine serum albumin, 25% formamide, 5× SSC and 0.1% SDS, at 42°C for 45 minutes. The slides were washed in sterile water and dried by centrifugation (3 minutes, 2,000 rpm). The labeled samples were boiled for 5 minutes, centrifuged for 1 minute, hybridized on the slide and placed in a hybridization chamber (Corning, Corning, NY, USA) for overnight incubation at 42°C. The slides were then washed for 5 minutes at 42°C with a solution containing 2× SSC and 0.1% SDS. An additional wash was performed at room temperature with a solution containing 0.1× SSC and 0.1% SDS, followed by three additional washes at room temperature in a 0.1× SSC solution.

Arrays were purchased from the Microarray Centre, University Health Network, Toronto, Canada, where PCR products for all ORFs were printed on each slide. Each ORF was printed in duplicated on the slide.

Images of arrays used for the experiment of normal germination in YPD medium (see Figure 2a for experimental design) were obtained using ScanArray 4000 scanner (Packard BioScience, MA, USA). Image analysis was performed using QuantArray version 3 software (PerkinElmer Life Sciences, Boston, MA, USA). Low-quality spots were discarded following detailed visual inspection along with other genes that were flagged by image analysis. The data were then transformed into log2 ratios, and normalized by subtracting the median. Values of replicate spots on the slides were averaged [44]. Images of arrays used for the experiment describing the response of spores to different components of the medium (see Figures 4a and 7a for experimental design) were obtained using Agilent's DNA microarray scanner. Image analysis was performed using SpotReader (Niles Scientific, CA, USA). Background intensity was subtracted using a Bayesian correction. The data were then transformed into log2 ratios and normalized by subtracting a Lowess regression followed by the median of each subarray [21]. The two spots corresponding to each gene were then averaged, and genes for which the two spots were significantly different were declared as 'missing values' (along with other genes that were flagged by image analysis or removed by manual inspection) [21]. The method of normalization did not significantly affect the results presented in this paper (Additional data file 2).

The data discussed in this publication have been deposited in NCBIs Gene Expression Omnibus (GEO) and are accessible through GEO Series accession number GSE7393 [45].

## Abbreviations

GFP, green fluorescent protein; GO, Gene Ontology; HA, hemagglutinin; ORF, open reading frame; SPO, sporulation medium; YPD, yeast extract/peptone/dextrose.

## Authors' contributions

DJS, DZ, GS and NB conceived and designed the experiments. DJS performed the experiments. DJS, GS and NB analyzed the data and wrote the manuscript. DZ provided detailed advice and commented on the text.

## Additional data files

The following additional data are available with the online version of this paper. Additional data file 1 includes Tables S1 to S7. Table S1 lists genes that were included in the different modules in Figure 2c. Table S2 lists GO annotations for genes that are induced during the first 15 minutes of spore germination. Table S3 lists GO annotations for genes that are repressed during the first 15 minutes of spore germination. Table S4 lists GO annotations for the two sub-groups of genes related to the G1/S module that are presented in Figure 9. Table S5 lists the yeast strains used in the present study. Table S6 lists PCR primers used in this study. Table S7 includes the composition of the media used in the present study. Additional data file 2 includes supplementary figures S1 to S4. Figure S1 shows mating of germinating cells before the appearance of their first buds. Figure S2 shows Cdc10-GFP protein localization in resting spores and at the beginning of spore germination. Figures S3 and S4 demonstrate that the usage of different normalization methods does not significantly affect the results presented in this paper.

## Supplementary Material

Additional data file 1 Table S1 lists genes that were included in the different modules in Figure 2c. Table S2 lists GO annotations for genes that are induced during the first 15 minutes of spore germination. Table S3 lists GO annotations for genes that are repressed during the first 15 minutes of spore germination. Table S4 lists GO annotations for the two sub-groups of genes related to the G1/S module that are presented in Figure 9. Table S5 lists the yeast strains used in the present study. Table S6 lists PCR primers used in this study. Table S7 includes the composition of the media used in the present study. Click here for file

Additional data file 2 Figure S1 shows mating of germinating cells before the appearance of their first buds. Figure S2 shows Cdc10-GFP protein localization in resting spores and at the beginning of spore germination. Figures S3 and S4 demonstrate that the usage of different normalization methods does not significantly affect the results presented in this paper. Click here for file
