# Supplementary material for: Spore germination in Saccharomyces cerevisiae: global gene expression patterns and cell cycle landmarks
Source: Genome Biol. 2007 Nov 14;8(11):R241. doi: 10.1186/gb-2007-8-11-r241 (PMC2258198; doi:10.1186/gb-2007-8-11-r241)
Supplement: Additional data file 1 — Table S1 lists genes that were included in the different modules in Figure 2c. Table S2 lists GO annotations for genes that are induced during the first 15 minutes of spore germination. Table S3 lists GO annotations for genes that are repressed during the first 15 minutes of spore germination. Table S4 lists GO annotations for the two sub-groups of genes related to the G1/S module that are presented in Figure 9. Table S5 lists the yeast strains used in the present study. Table S6 lists PCR primers used in this study. Table S7 includes the composition of the media used in the present study. [file gb-2007-8-11-r241-S1.doc]

Supplementary Table S1: Complete list of genes used in Figure 2c.

| Protein synthesis | RPL19A,RPS8A,RPL17A,RPS18B,RPS10A,URP1,CRY1,YS29B,RPL43A,YDL082W,YDL083C,  SOS2,SOS1,RPS18A,RPS13C,RPL45,RPL15A,RP51B,YDR450W,RPL27B,RPL35B,RPL15B,  RPS24EA,RPS8B,RPL17B,RPS26B,RPL32,RPL30A,RPL6A,CYH2,SUP44,SSM2,RPL9A,  RPS31A,YGR034W,RPL16A,RPS28A,RPL30B,YST1,RPL14B,URP2,RPL4A,RPL27,MAK18,  RPS7A,RPL5A,YIL052C,RPL13,UBI1,RPS25B,TIF2,YJL177W,RPS24A,RPS5,RPS7B,  RPL14A,YKL056C,RPS27A,RPL17,RPS25,TIF1,UBI2,RPL4B,RPL13A,YST2,YLR061W,  GRC5,UBI3,RPL35A,RPS33B,YLR325C,RPS31,YLR388W,RP10A,RPL16B,YML024W,  YML026C,RP10B,YL16A,BEL1,YMR142C,YMR242C,RPL9B,RP23,YNL096C,RPL41A,RPS3,  SSB2,RP28B,RPS16A,RPLA2,RPS21,RP28A,RPS16B,RPL25,TCM1,RPS30,RPS33A,  RPL37B,YOR293W,RPL18A1,RPS12,EGD1,YPL079W,YPL090C,RPL37A,SSM1,YPR102C,  RPS28B,YBR084CA,YER056CA,YFR031CA |
| --- | --- |
| rRNA processing | YDR101C,YDR496C,ROK1,YGR103W,YGR145W,YGR245C,DRS1,PWP1,YLR222C,DBP9,  YLR409C,YML093W,HAS1,YNL132W,YNL174W,YNL182C,YOR206W |
| Gluconeogenesis | YCR010C,ICL1,YFL030W,YGR067C,HXT5,YIL057C,MBR1,YKL187C,JEN1,PCK1,IDP2,  FBP1,CYB2,YMR107W,YMR206W,MLS1,YNL194C,YNL195C,GAC1,LEE1,PXA1,YPR030W |
| Stress | YDL204W,TPS2,YGL037C,STF2,CTT1,SOL4,GRE3,OM45,YJR096W,YKL091C,TFS1,  YLR251W,YLR252W,TSL1,YML128C,PGM2,YMR250W,YNL274C |
| TCA sub-cycle | CIT2,ACO1,IDH1,CIT1,YOR135C,IDH2,YPL087W,YPL135W,PEP4,YPR002W |
| oxydative phosphorylation | PET9,COR1,ATP1,ATP3,YBR183W,YBR230C,ATP16,COX9,INH1,SDH4,ATP5,ATP17,  QCR7,RIP1,YER053C,COX15,QCR6,COX4,COX13,CBP4,YGR182C,QCR9,COX6,QCR8,  CYC1,MIR1,ATP2,ATP7,MDH1,HAP4,SDH3,SDH1,MCR1,SDH2,COX12,YLR294C,  ATP14,COX8,NDI1,COX7,PBI2,COX5A,POR1,YNL100W,CIT1,CYT1,ATP4,ATP15,  YPR020W,QCR2,YHR001WA |
| proteosome subunits | PRE7,YBR062C,YBR173C,YTA5,RPN5,RPN4,YTA2,RPN8,PRE1,SUN2,PUP3,MPR1,  YFR010W,PRE4,NIN1,SCL1,SUG1,UFD1,PRE9,PHB2,PUP2,ARC15,PRE3,CAP1,  SBA1,YTA3,YKT6,YKR011C,YLR387C,YLR421C,GLO1,PRE8,PRE5,YNL155W,PRE6,  CRL13,RPN7,PRE10,PRE2,RPN6 |
| Mating | PRM9,YAR033W,FUS3,YBL062W,FIG1,YBR156C,YBR158W,YBR223C,YBR225W,  YBR226C,FUS1,KAR4,YCL074W,YCL075W,YCL076W,RVS161,FIG2,PCL2,RDI1,  AFR1,YDR124W,ECM18,YDR241W,YDR249C,PAM1,YDR309C,YDR340W,STE14,MFA1,  YER187W,STE2,YFL027C,YFL047W,AGA2,YGL052W,PRM8,IME4,YGL223C,GPA1,  STE12,YHR097C,CHS7,PRM2,YIL060W,YIL080W,YIL082W,YIL083C,PRM5,  YJL107C,PRM10,FAR1,ASG7,PGU1,GFA1,PGM1,PMU1,HYM1,YKL221W,KTR2,  YLR042C,MID2,SST2,PRP39,PRM6,KAR5,CIK1,FUS2,YNL042W,MSG5,INP52,  CHS1,YNL208W,PRM1,ERG24,AGA1,YOL095C,YOR129C,YOR343C,PRM4,PRM3,  YPL193W,KAR3,YML048WA,YIL082WA,YMR304CA |
| cell-cycle (G1) | RFA1,SEN34,HTB2,HTA2,YBL009W,POL12,HHF1,HHT1,YBR070C,YBR071W,RDH54,  RFC5,POL30,YBR089W,YCL022C,YCL024W,YCL061C,HCM1,MCD1,YDL018C,DUN1,  YDL163W,CDC9,ASF2,MSH6,PDS1,HTB1,HTA1,YDR279W,GIN4,YDR528W,MNN1,  PMI40,RNR1,RAD51,SSU81,ADK2,SMC1,CLB6,YGR151C,RSR1,YGR221C,YHR110W,  YHR127W,SPO16,YHR154W,YHR173C,IRR1,YIL132C,SRO4,SMC3,HPR5,ASF1,RFA3,  YJL181W,SWE1,POL32,PRI2,MIF2,HSL1,YKL108W,RAD27,YKR077W,YKR090W,KIM2,  SPA2,YLL022C,STU2,YLR049C,CDC45,YLR183C,TUB4,SPH1,YOX1,OGG1,CTF18,  YMR144W,SPT21,CLN1,HHF2,HHT2,PMS1,POL1,SPC98,YNL166C,BNI4,POL2,YIF1,  TOF1,RFA2,YNR009W,YOL007C,YOL017W,MSH2,BUB3,DHS1,CDC21,YOR114W,  YOR144C,NIP29,HHO1,RAD53,SVS1,IPL1,BBP1,CLN2,YPL267W,RLF2,CLB5,DPB2 |
| cell cycle G2/M | KIN3,YBL032W,CHS2,PHO3,CDC47,BUD3,YDR033W,SWI5,PMA1,ALK1,CDC20,DBF2,  CLB1,WSC4,MOB1,YIL158W,YJL051W,BUD4,YKL130C,YLR084C,ACE2,YLR190W,  YML034W,SUR7,YML058W,YML119W,CDC5,YMR032W,YMR215W,YNL057W,YNL058C,  YOL070C,HST3,YOR315W,YPL141C,KIP2,IQG1,CLB2,YCR024CA |

**Supplementary Table S2: Gene Ontology (GO) annotations for genes that are induced during the first 15 minutes of spore germination. The GO Term Finder tool found in the *Saccharomyces* Genome Database (SGD, www.yeastgenome.org) was used to search for significant shared GO terms used to describe genes that their log2 expression is induced by more than 4 fold 15 min after the induction of spore germination.**

|  | **GO_term** | **Frequency : Number of gene (out of 394 genes)** | **Genome Frequency: Number of genes (out of 7292 annotated genes)** | **Probability** |
| --- | --- | --- | --- | --- |
| 1 | ribosome biogenesis and assembly | 86 (21.8%) | 249 (3.4%) | 5.63E-43 |
| 2 | cytoplasm organization and biogenesis | 86 (21.8%) | 249 (3.4%) | 5.63E-43 |
| 3 | ribosome biogenesis | 67 (17.0%) | 210 (2.8%) | 2.77E-31 |
| 4 | rRNA processing | 54 (13.7%) | 171 (2.3%) | 4.93E-25 |
| 5 | rRNA metabolism | 56 (14.2%) | 250 (3.4%) | 4.55E-19 |
| 6 | RNA metabolism | 80 (20.3%) | 547 (7.5%) | 3.80E-16 |
| 7 | ribosome assembly | 27 (6.8%) | 64 (0.8%) | 5.80E-16 |
| 8 | RNA processing | 60 (15.2%) | 349 (4.7%) | 3.76E-15 |
| 9 | primary metabolism | 250 (63.4%) | 3203 (43.9%) | 5.16E-15 |
| 10 | ribosomal subunit assembly | 24 (6.0%) | 53 (0.7%) | 5.50E-15 |
| 11 | cellular process | 323 (81.9%) | 4759 (65.2%) | 1.55E-13 |
| 12 | cellular physiological process | 321 (81.4%) | 4720 (64.7%) | 1.91E-13 |
| 13 | physiological process | 323 (81.9%) | 4788 (65.6%) | 4.95E-13 |
| 14 | processing of 20S pre-rRNA | 20 (5.0%) | 44 (0.6%) | 9.75E-13 |
| 15 | metabolism | 258 (65.4%) | 3487 (47.8%) | 1.23E-12 |
| 16 | organelle organization and biogenesis | 109 (27.6%) | 1039 (14.2%) | 3.49E-12 |
| 17 | ribosomal large subunit assembly and maintenance | 18 (4.5%) | 40 (0.5%) | 1.58E-11 |
| 18 | macromolecule metabolism | 201 (51.0%) | 2548 (34.9%) | 4.60E-11 |
| 19 | cellular metabolism | 246 (62.4%) | 3397 (46.5%) | 1.87E-10 |
| 20 | cellular biosynthesis | 110 (27.9%) | 1126 (15.4%) | 2.08E-10 |
| 21 | biosynthesis | 115 (29.1%) | 1208 (16.5%) | 3.23E-10 |
| 22 | protein biosynthesis | 85 (21.5%) | 795 (10.9%) | 7.27E-10 |
| 23 | macromolecule biosynthesis | 88 (22.3%) | 857 (11.7%) | 2.40E-09 |
| 24 | protein complex assembly | 29 (7.3%) | 151 (2.0%) | 6.95E-09 |
| 25 | 35S primary transcript processing | 20 (5.0%) | 74 (1.0%) | 7.38E-09 |
| 26 | protein metabolism | 124 (31.4%) | 1449 (19.8%) | 3.45E-08 |
| 27 | cell organization and biogenesis | 131 (33.2%) | 1593 (21.8%) | 1.24E-07 |
| 28 | regulation of translational fidelity | 8 (2.0%) | 11 (0.1%) | 2.14E-07 |
| 29 | ribosomal large subunit biogenesis | 9 (2.2%) | 19 (0.2%) | 1.29E-06 |
| 30 | nucleobase, nucleoside, nucleotide and nucleic acid metabolism | 119 (30.2%) | 1526 (20.9%) | 9.45E-06 |

**Supplementary Table S3: Gene Ontology (GO) annotations for genes that are repressed during the first 15 minutes of spore germination.** The GO Term Finder tool found in *Saccharomyces* Genome Database (SGD, www.yeastgenome.org) was used to search for significant shared GO terms used to describe genes that their log2 expression is repressed by more than 4 fold 15 min after the induction of spore germination.

|  | **GO_term** | **Frequency : Number of gene (out of 102 genes)** | **Genome Frequency: Number of genes (out of 7292 annotated genes)** | **Probability** |
| --- | --- | --- | --- | --- |
| 1 | main pathways of carbohydrate metabolism | 11 (10.7%) | 73 (1.0%) | 7.81E-09 |
| 2 | aldehyde metabolism | 7 (6.8%) | 20 (0.2%) | 1.71E-08 |
| 3 | energy derivation by oxidation of organic compounds | 15 (14.7%) | 201 (2.7%) | 1.45E-07 |
| 4 | carbohydrate metabolism | 15 (14.7%) | 223 (3.0%) | 5.38E-07 |
| 5 | generation of precursor metabolites and energy | 15 (14.7%) | 233 (3.1%) | 9.28E-07 |
| 6 | cellular carbohydrate metabolism | 14 (13.7%) | 204 (2.7%) | 1.05E-06 |
| 7 | tricarboxylic acid cycle intermediate metabolism | 5 (4.9%) | 19 (0.2%) | 8.10E-06 |
| 8 | coenzyme metabolism | 10 (9.8%) | 121 (1.6%) | 8.39E-06 |
| 9 | organic acid metabolism | 15 (14.7%) | 302 (4.1%) | 2.06E-05 |
| 10 | carboxylic acid metabolism | 15 (14.7%) | 302 (4.1%) | 2.06E-05 |
| 11 | glyoxylate cycle | 3 (2.9%) | 4 (0.0%) | 2.72E-05 |
| 12 | citrate metabolism | 3 (2.9%) | 4 (0.0%) | 2.72E-05 |
| 13 | glyoxylate metabolism | 3 (2.9%) | 4 (0.0%) | 2.72E-05 |
| 14 | propionate metabolism | 3 (2.9%) | 5 (0.0%) | 5.26E-05 |
| 15 | pentose-phosphate shunt, oxidative branch | 3 (2.9%) | 5 (0.0%) | 5.26E-05 |
| 16 | cofactor metabolism | 10 (9.8%) | 153 (2.0%) | 6.07E-05 |
| 17 | alcohol metabolism | 10 (9.8%) | 161 (2.2%) | 9.22E-05 |

**Supplementary Table S4: Gene Ontology (GO) annotations for G1 genes** The GO Term Finder tool found in the *Saccharomyces* Genome Database (SGD, www.yeastgenome.org) was used to search for significant shared GO terms used to describe

a. G1/S genes expressed early in germination (genes 1-13 in Figure 9)

b. G1/S genes expressed late in germination (genes 81-98 in Figure 9)

**Supplementary Table S5**: Yeast strains used in this study

| **Strain** | **Genotype** | **Source** |
| --- | --- | --- |
| NKY1059 | *MATa lys2 ura3 leu2 ade2 his4 ho::hisG* | N. Kleckner |
| NKY561 | *MATα lys2 ura3::hisG leu2::hisG trp1::hisG ho::hisG* | N. Kleckner |
| D277  (NKY560-his3^) | *MATa ura3 trp1 leu2 lys2 ho::hisG his3::URA3*(GeneBlaster) | G. Simchen |
| DS6 | *MATa ura3 trp1 leu2 lys2 ho::hisG his3::URA3*  *CDC10-GFP(S65T)::HIS3MX* | This study |
| DS28 | *MATα lys2 ura3(PsI-SmaI)::hisG leu2::hisG trp1::hisG*  *ho::hisG CLB2-3HA::KanMX6* | This study |
| DS29 | *MATa lys2 ura3 leu2 ade2 his4 ho::hisG CLB2-3HA::KanMX6* | This study |
| DS1 | Diploid, constructed by mating of NKY1059 and NKY561 | This study* |
| DS35 | Diploid, constructed by mating of DS28 and DS29 | This study |
| DS38 | Diploid, constructed by mating of DS6 and NKY561 | This study |

* Reported also in Friedlander *et al*., (2006)

**Supplementary Table S6**: PCR primers used in this study

| **Primer** | **Sequence** |
| --- | --- |
| CDC10-F | 5'-GCAGTGGTTGGTTCTGAGAATG-3' |
| CDC10-R | 5'-ACTCTCGCCAATCCAGCAATAC-3' |
| CDC10-CHK | AAAGAATTGACAGCCCAACG |
| CLB2-F | GGTTAGAAAAAACGGCTATGATATAATGACCTTGCATGAAcggatccccgggttaattaa |
| CLB2-R | CGATTATCGTTTTAGATATTTTAAGCATCTGCCCCTCTTCgaattcgagctcgtttaaac |
| CLB2-CHK | TTTCGCATCAGAGACAGACG |
| Universal reverse | ATCACCTTCACCCTCTCCAC |
| U-CHK | TTAATTAACCCGGGGATCCG |

**Supplementary Table S7**: **Media used in the present study**

Synthetic minimal

2% glucose

0.17% yeast nitrogen base

0.5% ammonium sulfate

Supplemented with amino acids

YPD

1% yeast extract

2% bactopeptone

2% glucose

YPD+G418

G418 was added to YPD at a concentration of 0.2 mg/ml

Sporulation (SPO)

0.25% yeast extract

1.5% potassium acetate

0.05% glucose

Supplemented with all amino acids

For plates, agar was added to final concentration of 1.5%
