# Supplementary material for: Spore germination in Saccharomyces cerevisiae: global gene expression patterns and cell cycle landmarks
Source: Genome Biol. 2007 Nov 14;8(11):R241. doi: 10.1186/gb-2007-8-11-r241 (PMC2258198; doi:10.1186/gb-2007-8-11-r241)
Supplement: Additional data file 2 — Figure S1 shows mating of germinating cells before the appearance of their first buds. Figure S2 shows Cdc10-GFP protein localization in resting spores and at the beginning of spore germination. Figures S3 and S4 demonstrate that the usage of different normalization methods does not significantly affect the results presented in this paper. [file gb-2007-8-11-r241-S2.doc]

#

# Figure S1: Germinating cells can go through mating before the appearance of their first bud.

Purified *Cdc10-GFP* (DS38) spores were plated and synthetic minimal medium was added to allow spore germination. Images were taken at the indicated time points using Deltavision RT microscope system at 60x magnification.

**Figure S2: Cdc10-GFP protein localization in resting spores and at the beginning of spore germination.** Purified spores containing GFP tagged Cdc10 (prepared from strain DS38) were plated and synthetic minimal medium was added to allow spore germination. Time lapse microscopy was carried out using Deltavision RT microscope system with OAI Scan command and results deconvolved. Images were monitored at 100X magnification using FITC (excitation 490 and emission 526) filter. In yellow is the time following the addition of synthetic minimal medium.

#

# Figure S3: Comparing the two normalization methods that were used in this paper. Data obtained from the experiment of normal germination in YPD were normalized using both normalization methods [1, 2] and correlation coefficients (R) between the two sets were calculated. The distribution of R^2^ from linear fitting of the normalized data is presented.

**(a)**

**(b)**

**Figure S4: The similarity between gene expression following (a) incubation of spores in glucose, "nitrogen" or YPD (see Figure 4b) or (b) during spore germination of spores pre-incubated in glucose or "nitrogen" (see Figure 7b).**  Figures 4b and 7b were re-done using the same normalization method for all the data [2]. The different normalization methods do not significantly affect the results.
